# Usage and Daily Attrition of a Smartphone-Based Health Behavior Intervention: Randomized Controlled Trial

**DOI:** 10.2196/45414

**Published:** 2023-06-26

**Authors:** Erlendur Egilsson, Ragnar Bjarnason, Urdur Njardvik

**Affiliations:** 1 Department of Psychology University of Iceland Reykjavik Iceland; 2 Department of Pediatrics University of Iceland Reykjavik Iceland; 3 Faculty of Medicine University of Iceland Reykjavik Iceland

**Keywords:** mHealth intervention, mobile health, adolescent, attrition, mental health, physical activity

## Abstract

**Background:**

Although most adolescents have access to smartphones, few of them use mobile health (mHealth) apps for health improvement, highlighting the apparent lack of interest in mHealth apps among adolescents. Adolescent mHealth interventions have been burdened with high attrition rates. Research on these interventions among adolescents has frequently lacked detailed time-related attrition data alongside analysis of attrition reasons through usage.

**Objective:**

The objective was to obtain daily attrition rates among adolescents in an mHealth intervention to gain a deeper understanding of attrition patterns, including the role of motivational support, such as altruistic rewards, through analysis of app usage data.

**Methods:**

A randomized controlled trial was conducted with 304 adolescent participants (152 boys and 152 girls) aged 13-15 years. Based on 3 participating schools, participants were randomly assigned to control, treatment as usual (TAU), and intervention groups. Measures were obtained at baseline, continuously throughout the 42-day trial period (research groups), and at the trial end. The mHealth app is called SidekickHealth and is a social health game with the following 3 main categories: nutrition, mental health, and physical health. Primary measures were attrition based on time from launch, and the type, frequency, and time of health behavior exercise usage. Outcome differences were obtained through comparison tests, while regression models and survival analyses were used for attrition measures.

**Results:**

Attrition differed significantly between the intervention and TAU groups (44.4% vs 94.3%; χ^2^_1_=61.220; *P*<.001). The mean usage duration was 6.286 days in the TAU group and 24.975 days in the intervention group. In the intervention group, male participants were active significantly longer than female participants (29.155 vs 20.433 days; χ^2^_1_=6.574; *P*<.001). Participants in the intervention group completed a larger number of health exercises in all trial weeks, and a significant decrease in usage was observed from the first to second week in the TAU group (t_105_=9.208; *P*<.001) but not in the intervention group. There was a significant increase in health exercises in the intervention group from the fifth to sixth week (t_105_=3.446; *P*<.001). Such a significant increase in usage was not evident in the TAU group. The research group was significantly related to attrition time (hazard ratio 0.308, 95% CI 0.222-0.420), as well as the numbers of mental health exercises (*P*<.001) and nutrition exercises (*P*<.001).

**Conclusions:**

Differences in attrition rates and usage between groups of adolescents were identified. Motivational support is a significant factor for lowering attrition in adolescent mHealth interventions. The results point to sensitivity periods in the completion of diverse health tasks, and emphasis on time-specific attrition, along with the type, frequency, and time of health behavior exercise usage, is likely a fruitful avenue for further research on mHealth interventions for adolescent populations, in which attrition rates remain excessive.

**Trial Registration:**

ClinicalTrials.gov NCT05912439; https://clinicaltrials.gov/study/NCT05912439

## Introduction

Throughout the past decade, ownership of and access to smartphones and mobile devices have grown profoundly among adolescents and youth worldwide [1,2]. The growth has been such that smartphone ownership or access among US adolescents was 95% 4 years ago and had increased by 23% in the 4 years prior [1,2]. A similar development was observed in the majority of developed economies where adolescent smartphone access and ownership is above the 90th percentile [2]. Smartphones are so widely distributed and used that approximately 45% of adolescents spend nearly all waking hours online [3]. However, modest projections of daily usage indicate that many spend way less time online each day, though it is usually more than 4 hours [4-6].

Widespread smartphone usage in adolescent and youth populations has been extensively covered, but a more positive side to mobile usage is that a significant proportion of adolescents seek health information and clinical help online through their mobile devices, providing ample opportunities to reach at-risk adolescents with science-based methods focusing on health improvement [7-9]. Health problems (ie, mental health and lifestyle diseases) disproportionally burden lower socioeconomic status groups as well as diverse minority groups, and smartphones could become a vital tool for eliminating such disparities since smartphone access and ownership are not related to socioeconomic status, gender, or race in diverse economies [2,10,11]. The mobile health (mHealth) market is steadily becoming saturated with apps, and the yearly increase in the number of apps available has skyrocketed in recent years, with an estimated 350,000 mHealth apps currently on the market [12]. However, only 8% of adolescents seem to use health apps to improve their health, highlighting the apparent gap between easy access, extensive daily usage, and lack of interest in mHealth apps among adolescents [13].

Lack of physical activity has been labeled a global pandemic and has been reported as the fourth leading global cause of death [14]. Physical inactivity increases the risk of lifestyle diseases, such as heart disease, type 2 diabetes, and cancer, resulting in over 5 million annual global deaths [15]. Further, the estimated annual financial burden of physical inactivity is nearly USD 54 billion in health care costs around the world [16]. There seems to be a drop in physical activity in adolescence, and a large number of adolescents are under the recommended physical activity levels provided by the World Health Organization (WHO) [17-19]. Lack of sufficient physical activity tends to continue into adulthood, and research suggests that the majority of adolescents in the European Union do not even reach 30% of the recommended daily physical activity [19-21]. Further, adolescents seem to have the unhealthiest diet of all age groups, and they are particularly susceptible to weight gain [22]. Research has repeatedly revealed a significant relationship between nutritional behavior and physical activity in terms of weight management [23]. A tremendous increase in global adolescent obesity has been witnessed in the past decades, and the prevalence, for instance, has tripled since 1975 [24]. Cost-effective interventions to increase physical activity and improve nutritional behaviors in adolescent populations are therefore urgently needed.

Physical inactivity and inadequate nutritional habits are often interrelated to disabling emotional problems, and integrated strategies should include all 3 pillars to improve physical as well as mental well-being in adolescent populations. mHealth interventions targeting disabling emotional problems in adolescent populations have revealed encouraging outcomes, despite the fact that attrition rates in these interventions are generally high [11,25-30]. Varying definitions of attrition have complicated research on this topic, but attrition is defined as leaving treatment before obtaining a required level of improvement or completing intervention goals [31-33]. Research on mental mHealth interventions among adolescents has frequently lacked detailed time-related attrition data alongside accurate definitions and analysis of attrition reasons, though recent studies show promise in that regard [11,30,34]. Attrition is regularly reported at 2 distinct points of time, that is, intervention start and intervention end. A continuous measure of usage versus nonusage in mHealth interventions for adolescents while simultaneously obtaining detailed usage data to prevent or delay exact times of attrition in future interventions, would perhaps be an improved representation of attrition [35].

Increased knowledge on the actual attrition factors and patterns associated with mHealth interventions in adolescent populations is urgently needed. Obtaining a better understanding of how motivational support motivates adolescents to use mHealth apps and why adolescents maintain or lose interest in using these apps to improve their health is of vital importance. Motivational support in mHealth interventions, defined as strategies to enhance motivation and counter attrition to overcome behavior change barriers, often includes goal-setting, feedback, social support, and rewards [36,37]. Systematic reviews examining possible drivers behind usage point to group and task customization, localization, functional user support, gamification of health tasks, and immediate visual but simplified feedback on user action, while gender-related motivational support features could be contributing factors [36-38]. The timing of tailored motivational support, through just-in-time adaptive interventions, should be considered as well when implementing adolescent mHealth interventions, since time-based individualization could counter high attrition rates [35,39]. Given the magnitude of reported health problems among adolescents and lack of cost-effective health behavior interventions specifically developed for adolescent populations, the need for a better understanding of attrition reasons in adolescent mHealth interventions is large. The study aimed to (1) seek a richer understanding of continuous attrition rates for an mHealth intervention in an adolescent population and the effects motivational support has on attrition rates, and (2) examine the effectiveness of the intervention with the aim to increase daily mental, nutritional, and physical health behaviors.

## Methods

### Participants

The study included 304 individuals (152 girls and 152 boys) aged 13 to 15 years attending 1 of 3 public schools for children and adolescents in the greater capital area of Iceland. The mean age at baseline measurement was 13.70 (SD 0.83) years. All children attending the highest 3 classes (8th to 10th classes) in the 3 participating public elementary schools in Iceland were eligible to participate (n=661; male-to-female ratio of 313:348). All children in public schools in the municipality are equipped with an iPad from 10 years of age. The exclusion criterion was the diagnosis of a severe disorder of intellectual development or a physical, developmental, or mental illness significantly restricting the ability to use mobile apps. No participant was excluded from the study based on this exclusion criterion. Research specifications and an introduction to the app were sent via email to the parents and legal caretakers of all eligible participants through school officials, along with a confirmative survey link. If the link was answered, it provided confirmation for informed consent. Adolescents with informed consent from parents or legal caretakers were invited to take part in the study through a confirmative survey link.

### Ethics Approval

The study was approved by the National Bioethics Committee of Iceland (license number: VSNb2015060065/03-01).

### Measurements

The amount, time, and frequency of daily health activities measured through completion of in-app exercises, quality of sleep and energy levels, self-reported stress levels, and gratitude levels were primary outcome measures. The Cronbach α for the current sample was .920 for all self-reported health tasks within the app.

Anxiety and depressive symptoms were assessed using the Revised Children’s Anxiety and Depression Scale (RCADS), a self-report assessment tool for children and youth. The scale involves a 4-point Likert scale, spans 47 questions, and is divided into 6 subscales (separation anxiety symptoms, general anxiety symptoms, obsessive-compulsion symptoms, social anxiety symptoms, panic symptoms, and depression symptoms). A *T*-score over 65 marks the clinical cutoff point. The inventory’s psychometrics have been studied with acceptable findings in both US and Icelandic pediatric populations [40,41]. The Cronbach α for the current sample was .958.

The General Self-Efficacy Scale (GSE), a 10-item self-report questionnaire with the total score ranging from 10 to 40, was used to measure self-efficacy levels, with a higher score indicating higher self-efficacy [42]. Acceptable psychometric properties for the questionnaire have been obtained, and it has been used globally in youth populations [43]. The Cronbach α for the current sample was .937.

The *BEARS* sleep screening algorithm was used to evaluate participants’ sleep problems. It is a sleep screening instrument for children from 2 to 18 years old, and is divided into 5 sleep domains (bedtime problems, excessive daytime sleepiness, awakenings during night, regularity and duration of sleep, and snoring) [44]. The algorithm’s psychometrics have been studied with acceptable findings in pediatric populations [45]. The Cronbach α for the current sample was .769.

### mHealth App

**Figure 1:**
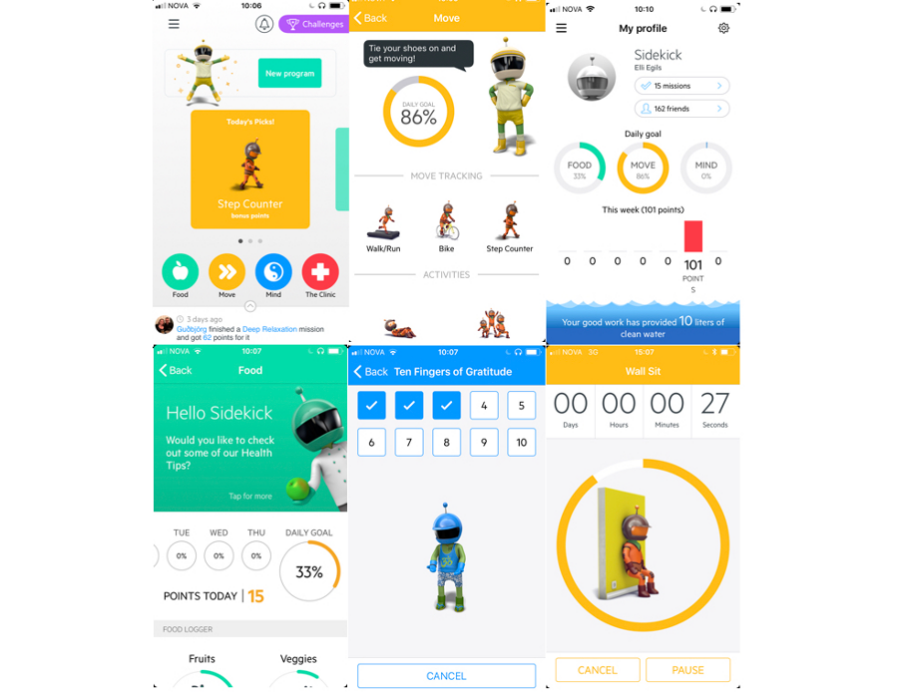
Overview of app functions and categories.

The app is called SidekickHealth and has been described in the research group’s previous work [35]. SidekickHealth was initially developed through multiple focus group studies among both Icelandic elementary school students and adolescents in the obesity clinic at the Landspitali University Hospital in Iceland to incorporate the target groups’ needs and opinions. Based on results from focus group studies and design advisors, the app took the form of a social health game (Figure 1). Functionality of the app evolves around motivational support to help the user set goals and complete health tasks (gamification of tasks) in the following 3 main categories: food and drink (eg, vegetable and water intake, consumption of fruits, and avoiding sugary soda or energy drinks), physical activity (eg, body weight exercises, logged minutes of sports activity, GPS-based biking, walking, and running), and mental health exercises (eg, reducing stress, exercising gratitude, and improving sleep habits). By completing health tasks that are labeled missions and participating in friendly competitions with peers, users earn points (called “kicks”) and badges providing altruistic rewards (eg, liters of water for children in need or polio vaccines that are sent in their name to children in need through UNICEF). A visual representation of the user’s performance is provided in different categories. Keeping the app fun, entertaining, and easy to use is of integral importance and was a strong focus point throughout the developmental phases (Multimedia Appendix 1). The smartphone app operates on the Android and iOS platforms. The app’s function focuses on education and simple health behavior changes through the benefits of increased physical activity and mental health exercises, as well as a healthy diet, portion sizing, and appetite awareness training. Appetite awareness training is a behavior modification tool that has, for instance, been used in obesity treatment and encourages overweight/obese children and youth to consume food and drink in response to internal appetite cues. It has shown promise for the treatment of overweight and obese children and teenagers, and has been visually developed as an individual mission in the app’s nutrition category [46,47]. Participants in the intervention arm were randomly assigned to groups consisting of 8 individuals that collectively and individually competed in point collection through completion of in-app health tasks. In the beginning of each of the trial’s 6 weeks, the intervention group received in-app messages where a new weekly competition (both individual and group levels) with altruistic rewards was introduced. In weeks 2 to 6, altruistic rewards for the past week’s efforts were also handed out. Winners of competitions received confirmation that UNICEF had sent polio vaccines to children in need. Further, through completion of in-app health exercises, participants collected liters of water that were sent in their name to children in need through UNICEF. The total cost for the altruistic rewards, paid for by the first author, throughout the treatment period was roughly US $68 (56 US cents per participant).

### Procedure

This study was a randomized controlled study. Group randomization was used to divide the 3 participating schools into control, treatment as usual (TAU), and intervention groups. Measures were obtained at baseline and 42 days later. Participants in both the TAU and intervention groups received an approximately 10-minute-long introduction regarding the study specifications and the app. The control group received no further contact, access to the app, or information until study-end questionnaire measures. Participants in the intervention group were randomly assigned to teams consisting of 8 individuals that collectively and individually competed in point collection through completion of in-app health tasks. Participation in the TAU and intervention groups was defined as downloading the SidekickHealth app and completing at least 3 health exercises within it. Exercise time was defined as the timestamp on completion of the exercise within any of the 3 types of exercise categories (physical activity, nutrition, and mental health) in the app. Exercise frequency referred to how often a given exercise was completed by a participant. Attrition time was defined as the timestamp of the last completed health exercise within the SidekickHealth app throughout the intervention period. The procedural difference between the TAU and intervention groups is related to motivational support. The intervention group received motivational support in the form of weekly individual and group feedback on usage, participation in friendly health task competitions, and weekly altruistic rewards for usage. Participants in the TAU group used the app individually throughout the trial period without any motivational support. A flowchart of participation is displayed in Multimedia Appendix 2.

### Statistical Analysis

The descriptive characteristics of participants along with attrition reasons are reported. Pearson correlation coefficients, independent samples *t*-tests, repeated measures ANOVA with adjusted alpha levels, and χ^2^ tests were used to measure mean differences in primary and secondary outcome measures from baseline to the trial end within and between research groups. Kaplan-Meier survival analysis plots and log-rank tests were used to assess the attrition time and possible significant differences between and within research groups [48,49]. The trial start was defined as the time of the first in-app health exercise completion, and the trial period was 6 weeks (42 days) from that moment. Attrition, or the event, was defined as the time of the participant’s last completed health exercise in the SidekickHealth app. Participant cases were evaluated as censored when the app was still being used 42 days after the study start. Cox proportional hazard regression models with interacting covariables using research groups as clusters were used to examine attrition prediction based on usage of in-app health exercises for the time, type, and frequency of exercises, as well as sociodemographic variables (age and gender) [50]. Significance was defined as a *P* value <.05. Data were analyzed using IBM SPSS Statistics, Release Version 29 (IBM Corp).

## Results

**Figure 2:**
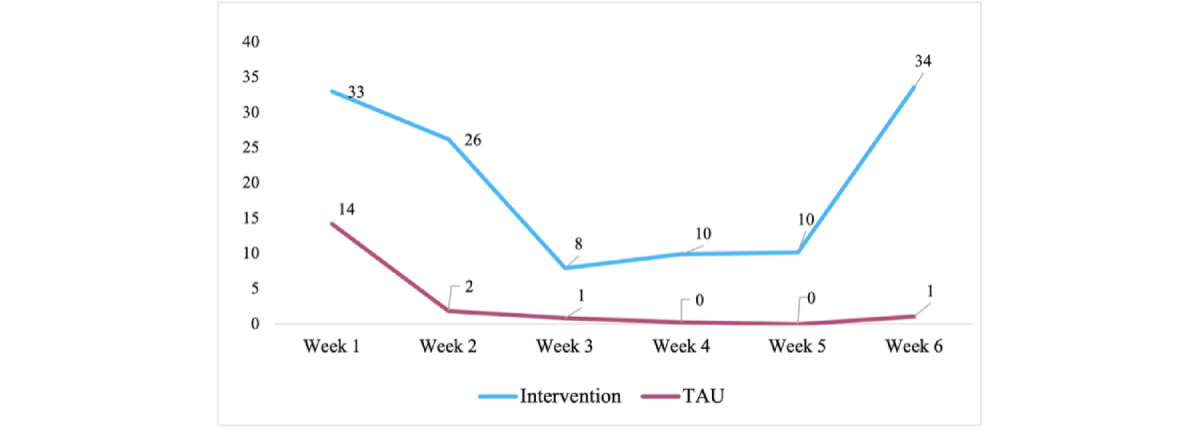
Mean weekly in-app exercises. TAU: treatment as usual.

Among all invited participants with parental or caretaker consent to participate (N=451), 304 (67.41%) individuals took part in the study. Participants who did not answer questionnaires at the study end were excluded. Participant characteristics are presented in Table 1. Logged data revealed broad differences in app usage among participants as shown in Figure 2. There was a significant difference in the mean number of health exercises completed by participants, where individuals in the intervention group (mean 120.869, SD 32.434) completed on average roughly 6 times as many exercises as individuals in the TAU group (mean 18.341, SD 31.802) over the study period (t_221_=−3.00; *P*<.001). When logged health exercises on the first day of the study period were examined, the results showed that the difference between the intervention group (mean 16.835, SD 21.820) and TAU group (mean 8.100, SD 7.237) was less extensive but still significant (t_221_=−2.12; *P*=.04).

**Figure 3:**
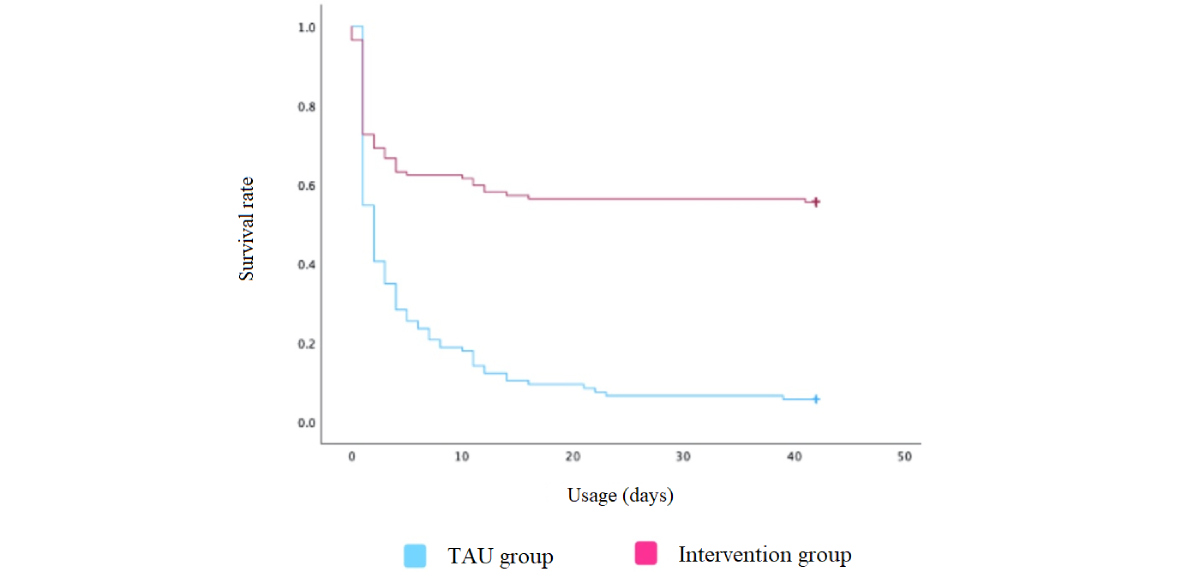
Forms of attrition. TAU: treatment as usual.

**Figure 4:**
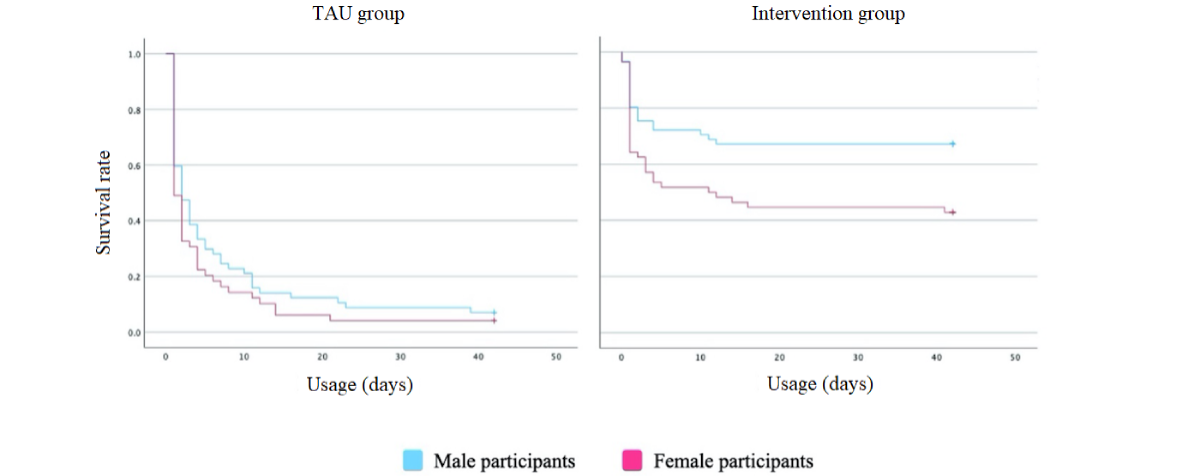
Gender-based attrition in the TAU and intervention groups. TAU: treatment as usual.

Forms of attrition over the 42-day study period are shown in Figure 3. Significant differences in completion rates were evident in log-rank tests between the intervention group and TAU group (χ^2^_1_=61.220; *P*<.001). Among participants in the TAU group, the mean survival time was 6.286 days (95% CI 4.304-8.277), while among participants in the intervention group, the mean survival time was 24.975 days (95% CI 21.452-28.518). Log-rank tests revealed significant differences in completion rates between male and female participants in the intervention group (χ^2^_1_=6.574; *P*<.001). In the intervention group, the mean survival time among male participants was 29.155 days (95% CI 24.519-33.812) and among female participants was 20.433 days (95% CI 15.301-25.558). Such differences were not evident in log-rank tests in the TAU group (χ^2^_1_=1.570; *P*=.21). Figure 4 presents Kaplan-Meier plots for gender-based attrition in the TAU and intervention groups.

There was a significant difference between groups in the average number of in-app health exercises in all weeks (Table 2). There was a significant mean drop (mean 12.347, SD 13.803) in usage in the TAU group from the first week to the second week (t_105_=9.208; *P*<.001). Even though there was a drop in usage in the intervention group between the first and second weeks of the trial (mean 6.798, SD 37.481), the difference was not significant (t_105_=1.959; *P*=.06). There was however a significant increase (mean 22.904, SD 71.721) in the average individual in-app health exercises completed by the intervention group from the fifth week to the sixth and last week of the trial (t_105_=3.446; *P*<.001). Such a significant increase in usage was not evident in the TAU group.

No significant gender differences were found in the average weekly in-app exercise frequency within research groups (Table 3).

**Figure 5:**
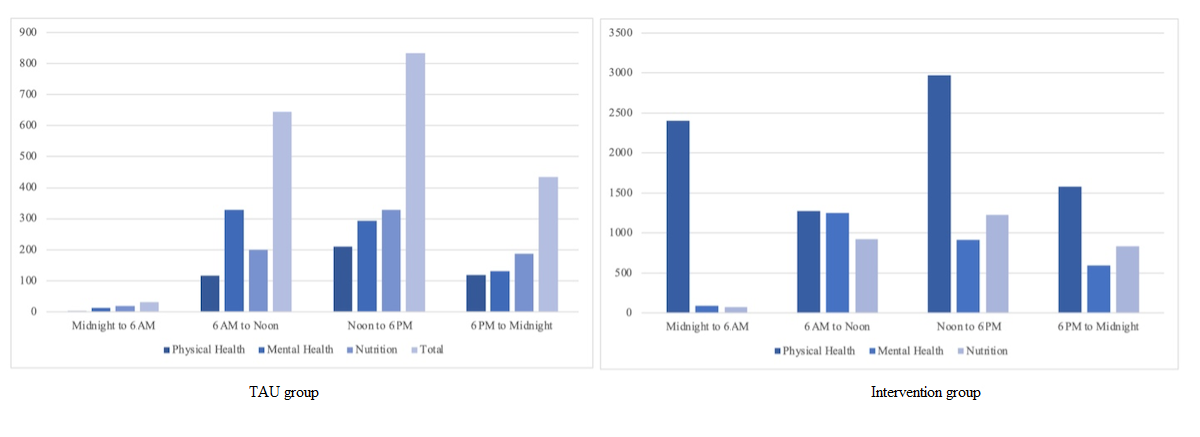
Time-based exercise categories in the TAU and intervention groups. TAU: treatment as usual.

When exercise time was compared with the exercise category, the results revealed significant differences within both the intervention group (χ^2^_6_=2162.559; *P*<.001) and the TAU group (χ^2^_6_=69.372; *P*<.001). Differences in usage based on exercise time and exercise type are presented in Table 4 and Figure 5.

Results from the Cox proportional hazard regression models are shown in Multimedia Appendix 3. The research group that participants were assigned to (hazard ratio 0.308, 95% CI 0.222-0.420) was significantly related to attrition (*P*<.001), as well as the numbers of mental health exercises (*P*<.001) and nutrition exercises (*P*<.001) completed in the app by participants in both research groups. Participants in the TAU group (hazard ratio 0.387, 95% CI 0.201-0.748) who completed an in-app health exercise between day 2 and day 6 of the trial were found to be significantly more likely to finish (*P*=.03). Such significant differences were not found in the intervention group. Further, the numbers of health exercises completed in the app in the first week (*P*<.001), second week (*P*<.001), and last week (*P*<.001) of the trial were significantly related to survival rates in the TAU group. Similar significant differences were not evident in the intervention group. All types of health exercises completed in the app were also significantly related to attrition in the TAU group, although such differences were not found among intervention group members. Anxiety, depression, and self-efficacy measures between research groups are shown in Multimedia Appendix 4.

**Table 1:** Baseline participant characteristics.

Characteristic	Control group (n=81)	TAU^a^ group (n=106)	Intervention group (n=117)
Age (years), mean (SD)	13.72 (0.45)	13.14 (0.51)	13.50 (0.63)
Male:female ratio	34:47	57:49	61:56
Disabling sleep problems, n (%)	32 (39.5)	23 (21.7)	38 (32.5)
Clinical anxiety symptoms, n (%)	18 (22.2)	10 (9.4)	14 (12.0)
Clinical depression symptoms, n (%)	12 (14.8)	7 (6.6)	7 (6.0)
General self-efficacy score, mean (SD)	17.90 (5.89)	16.17 (5.71)	18.14 (5.04)

^a^TAU: treatment as usual.

**Table 2:** Weekly comparison of usage and attrition.

Week	In-app health exercises completed, n			TAU^a^ group usage, mean (SD)	Intervention group usage, mean (SD)	*P* value
	Overall	Male	Female			
1	5369	3531	1838	14.20 (17.34)	33.03 (72.38)	<.001
2	3265	2514	751	1.85 (8.71)	26.23 (90.61)	<.001
3	1016	548	468	0.84 (5.38)	7.92 (26.71)	<.001
4	1192	803	389	0.24 (1.28)	9.97 (34.08)	<.001
5	1223	891	332	0.00 (0.00)	10.45 (60.09)	<.001
6	4029	2889	1140	1.20 (6.99)	33.35 (110.99)	<.001
Overall	16,094	11,176	4918	18.32 (27.44)	120.96 (350.09)	<.001

^a^TAU: treatment as usual.

**Table 3:** Weekly average in-app health exercise frequency by gender.

Week and group		Male, mean (SD)	Female, mean (SD)	*P* value
**Week 1**				
	TAU^a^	16.19 (19.36)	11.88 (14.50)	.20
	Intervention	42.75 (97.75)	22.43 (20.12)	.13
**Week 2**				
	TAU	3.02 (11.71)	0.49 (1.56)	.14
	Intervention	38.89 (72.64)	12.98 (23.79)	.13
**Week 3**				
	TAU	0.07 (0.42)	1.73 (7.84)	.11
	Intervention	8.92 (22.29)	6.84 (30.99)	.68
**Week 4**				
	TAU	0.37 (1.70)	0.08 (0.45)	.25
	Intervention	12.84 (30.06)	6.88 (26.09)	.35
**Week 5**				
	TAU	0.00 (0.00)	0.00 (0.00)	N/A^b^
	Intervention	14.61 (62.43)	5.93 (12.73)	.44
**Week 6**				
	TAU	1.02 (4.66)	1.41 (9.02)	.78
	Intervention	46.41 (77.42)	19.13 (43.32)	.19
**All weeks**				
	TAU	20.67 (33.60)	15.59 (17.85)	.35
	Intervention	163.90 (271.22)	74.18 (109.50)	.08

^a^TAU: treatment as usual.

^b^N/A: not applicable.

**Table 4:** Frequency of exercise categories at different daily times.

Group and exercise category		Midnight to 6 AM, n (%)	6 AM to noon, n (%)	Noon to 6 PM, n (%)	6 PM to midnight, n (%)
**TAU^a^ group**		32 (100)	644 (100)	833 (100)	435 (100)
	Physical health	1 (3.1)	116 (18.0)	210 (25.2)	119 (27.4)
	Mental health	12 (37.5)	329 (51.1)	294 (35.3)	130 (29.9)
	Nutrition	19 (59.4)	199 (30.9)	329 (39.5)	186 (42.7)
**Intervention group**		2575 (100)	3446 (100)	5113 (100)	3008 (100)
	Physical health	2407 (93.5)	1275 (37.0)	2970 (58.1)	1576 (52.4)
	Mental health	90 (3.5)	1248 (36.2)	912 (17.8)	599 (19.9)
	Nutrition	78 (3.0)	923 (26.8)	1231 (24.1)	833 (27.7)
**Overall**		2607 (100)	4090 (100)	5946 (100)	3443 (100)
	Physical health	2408 (92.4)	1391 (34.0)	3180 (53.5)	1695 (49.2)
	Mental health	102 (3.9)	1577 (38.6)	1206 (20.3)	729 (21.2)
	Nutrition	97 (3.7)	1122 (27.4)	1560 (26.2)	1019 (29.6)

^a^TAU: treatment as usual.

## Discussion

The focus of this study was on time-specific attrition in an adolescent mHealth intervention. We hoped to build on previous work while focusing on the type, frequency, and time of usage in order to better understand why adolescent attrition from mHealth interventions is generally as excessive as it is, with a market saturated with roughly 350,000 mHealth apps adolescents seem reluctant to engage in [12,35]. The results showed that research groups are related to time of attrition (hazard ratio 0.308, 95% CI 0.222-0.420), and attrition differed between the TAU group (94.3%) and intervention group (44.4%). Attrition was somewhat higher than in our previous study, where attrition was in the 35th percentile. The difference in attrition rates between research groups was however vast, and the only distinction in program setup was motivational support in the form of weekly feedback on individual progress through the app, friendly health task competitions at the individual and group levels, and small altruistic rewards for completion of health tasks or active participation in competitions. The app was the same, but support through program setup differed. It is interesting that the altruistic reward cost per participant was only 56 US cents but still seemed to contribute strongly to increased usage and participation.

Completion rates differed greatly between research groups as did days of active usage since the number of usage days in the intervention group was nearly 25 (95% CI 21.452-28.518) and that in the TAU group was approximately 6 (95% CI 4.304-8.277). Differences in completion rates were therefore evident between the intervention group (55.6%) and TAU group (5.7%), and gender differences in completion rates were also observed in the intervention group but not in the TAU group. Male participants (95% CI 24.519-33.812) completed in-app health tasks longer than female participants (95% CI 15.301-25.558). This gender difference was not evident in our prior research but has been observed in adult populations and should be examined in future research hoping to explain attrition factors in adolescent mHealth interventions [38]. A deeper gender-based exploration into motivational support could be a promising avenue for further research on the matter, particularly how altruistic rewards and competitive intervention features facilitate motivational support between genders.

Broad differences in the completion of health exercises were evident between groups since the intervention group completed on average roughly 6 times as many health exercises as the TAU group throughout the trial period. It is somewhat interesting that this difference was only 2-fold on the first day of the trial, suggesting that participants in the TAU group did not lack usage motivation at the beginning of the trial but were simply not supported to keep on using the app. This was more evident when average weekly health exercise frequency was examined since there was a decrease in usage between the first and second weeks among participants in the TAU group, while such a difference was not evident among participants in the intervention group. In fact, intervention group members completed on average more health exercises in all 6 weeks of the trial than their peers in the TAU group. An interesting finding is that usage increased from the fifth week of the trial to the last one among intervention group members but not among TAU group members, which is thought to be related to motivational support features as well as the altruistic reward setup at the end of the trial’s sixth week, and warrants further research.

There seemed to be different daily sensitivity periods for increased frequency of health task completion in different health categories. Adolescents in both research groups completed most of the physical activity exercises within the app from noon to 6 PM. The same applied to nutrition exercises in both groups. However, when it came to the frequency of mental health exercises, adolescents in both groups tended to do them from 6 AM to noon. Further, results from regression models indicated that the frequency of mental health exercises as well as nutrition exercises completed in the app by participants in both groups was related to delayed attrition. Physical activity exercises did not show such effects, possibly because those participants who did few exercises and were likely to drop out mainly used the physical activity category. Adolescents in the TAU group who completed an in-app health exercise between day 2 and day 6 of the trial were also found to be more likely to finish. This was not evident in the intervention group. These results imply that there are sensitivity usage periods that differ between the types of health behaviors adopted by adolescents in mHealth interventions and highlights the need for the development of just-in-time adaptive interventions in the future to hamper attrition and hopefully increase the frequency of health behavior exercises.

Taken together, the aim of this study was to examine time-specific attrition rates in an mHealth intervention for adolescents and hopefully increase our understanding of attrition in this group through a focus on the type, frequency, and time of health behavior exercise usage. Attrition between research groups was vastly different, and motivational support seems to be of vital importance to lower attrition in future mHealth interventions, specifically for adolescents in different age groups. Further research on how specific motivational support features in adolescent mHealth interventions function to lower attrition rates and how they affect usage patterns is evidently needed.

The limitations of this research include randomization factors, since randomization was between elementary schools rather than on an individual level to prevent contamination effects. Another limitation was related to the initial difference in usage between research groups. The data collection period was 6 weeks, and further research on the matter should include a prolonged research period with added randomization efforts to level usage between research groups, along with a 3-month follow-up to track usage and sustained gains from motivational support features. The generalizability of findings in adolescent mHealth studies to wider populations can be questionable, and this study is no exception (for instance, the function of altruistic reward schemes and competitive features in diverse cultural settings). The study’s foremost strength lies in added knowledge to limited research on attrition rates and patterns in adolescent mHealth interventions. Additional strong points are related to the methodological approach. Continuous data collection throughout the trial period, efforts to accurately describe time-based attrition rates through survival analysis, and use of a relatively large sample size (n=304) are regarded as strengths.

## Appendix

Multimedia Appendix 1 App function.

Multimedia Appendix 2 Study flowchart.

Multimedia Appendix 3 Regression model.

Multimedia Appendix 4 Anxiety, depression, and self-efficacy measures between research groups.

Multimedia Appendix 5 CONSORT-EHEALTH checklist.

## Abbreviations

mHealth: mobile health
TAU: treatment as usual
