# Supplementary figures and images for: Usage and Daily Attrition of a Smartphone-Based Health Behavior Intervention: Randomized Controlled Trial
Source: JMIR Mhealth Uhealth. 2023 Jun 26;11:e45414. doi: 10.2196/45414 (PMC10337294; doi:10.2196/45414)

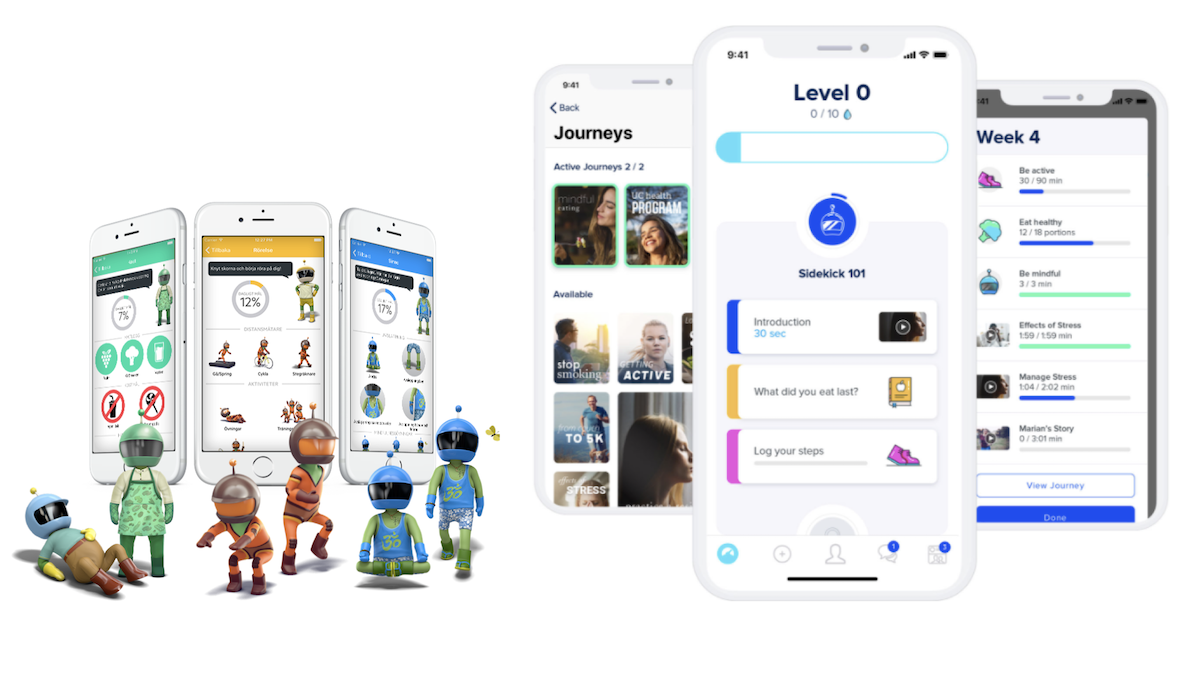

Supplement: Multimedia Appendix 1 [file mhealth_v11i1e45414_app1.png]

## Multimedia appendix 2. RCT intervention flow chart

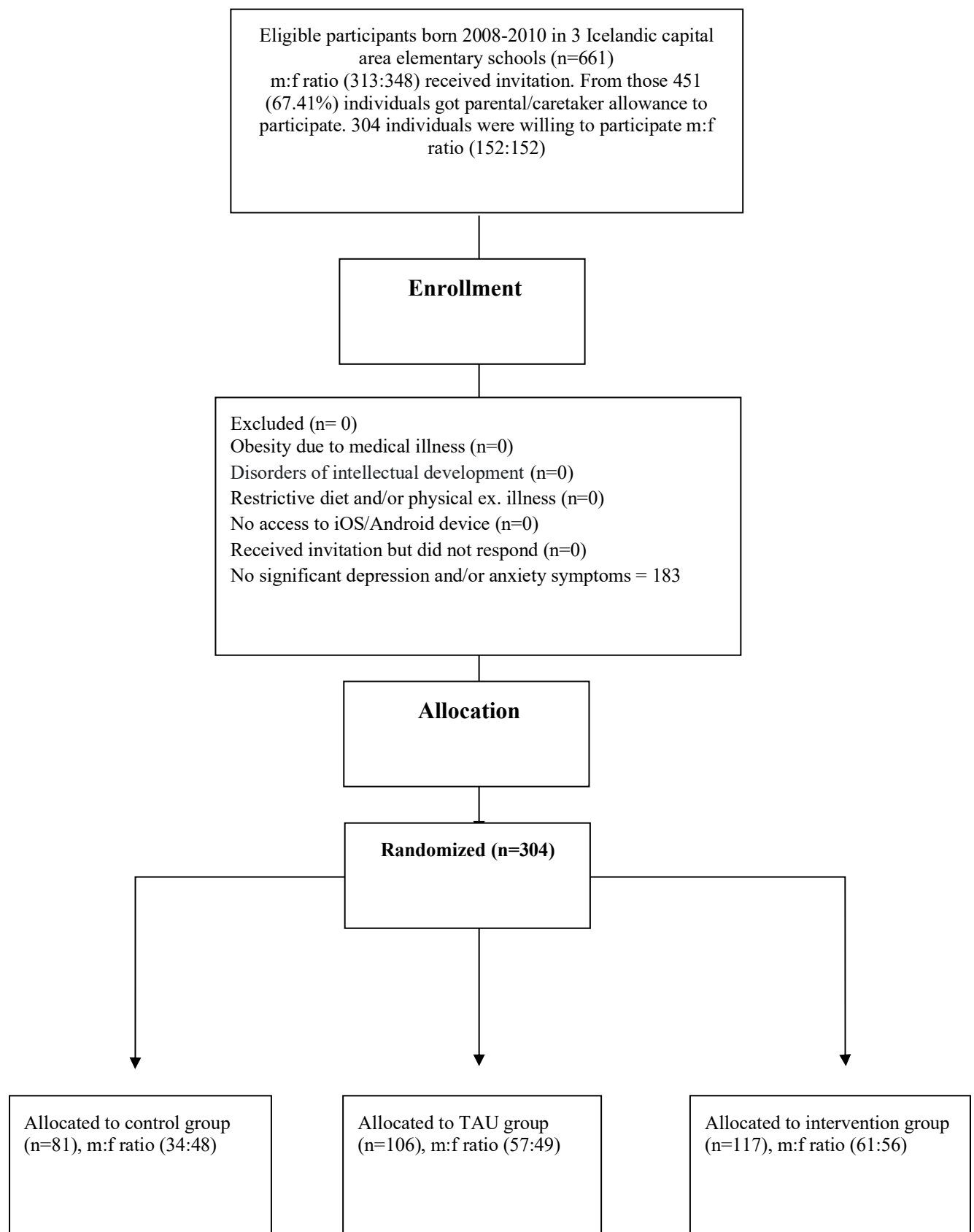

Supplement: Multimedia Appendix 2 [file mhealth_v11i1e45414_app2.pdf]
