# Supplementary material for: Usage and Daily Attrition of a Smartphone-Based Health Behavior Intervention: Randomized Controlled Trial
Source: JMIR Mhealth Uhealth. 2023 Jun 26;11:e45414. doi: 10.2196/45414 (PMC10337294; doi:10.2196/45414)
Supplement: Multimedia Appendix 3 [file mhealth_v11i1e45414_app3.pdf]

Multimedia Appendix 3. Results of Cox proportional hazard regression model

		Coefficient	HR (95% CI)	*P* value	Coefficient	HR (95% CI)	*P* value
**Research Group**		.18	.31 (.22 - .42)	.000			
**Exercise Type**							
	Number of Physical Health Exercises	.24	.96 (.92-.1.00)	.067			
	Number of Mental Health Exercises	.20	.93 (.90-.97)	.000			
	Number of Nutrition Exercises	.02	.95 (.92-.98)	.000			
			**TAU Group**			**Intervention**	
**Sociodemographics**							
	Gender	.23	.92 (0.58-1.46)	.728	.33	1.66 (.87-3.15)	.118
	Age	.21	.97 (.64-1.46)	.882	.24	1.40 (0.87-2.26)	.157
**Frequency of Health Exercises**							
	Exercised W1 (After 1st Day)	.34	.39 (.20-.75)	.009	.43	.57 (.25-1.33)	.191
	Number of Exercises in Week1	.52	5.69 (2.06-15.71)	.000	.13	1.15 (.89-1.49)	.284
	Number of Exercises in Week2	.51	4.44 (1.63-12.11)	.000	.16	1.16 (.84-1.58)	.367
	Number of Exercises in Week3	.02	1.00 (.95-1.04)	.821	.04	1.00 (.94-1.08)	.921
	Number of Exercises in Week4	.96	2.94 (0.45-19.20)	.258	.13	.98 (.76-1.28)	.904
	Number of Exercises in Week5				.44	1.10 (.46-2.62)	.839
	Number of Exercises in Week6	5.68	.00 (.00-.02)	.000	.28	.81 (.46-1.41)	.445
**Exercise Type**							
	Number of Physical Health Exercises	.57	.13 (.04-.41)	.000	.16	.93 (.69-1.26)	.641
	Number of Mental Health Exercises	.53	.16 (.06-.44)	.000	.14	.83 (.63-1.10)	.193
	Number of Nutrition Exercises	.53	.17 (.06-.47)	.000	.15	.88 (.66-1.18)	.396
**Time of Exercise**							
	Number of Exercises from Midnight to 6AM	.26	.71 (.43-1.19)	.201	.15	.92 (.68-1.23)	.566
	Number of Exercises from 6AM to Noon	.07	1.08 (.93-1.24)	.309	.06	.94 (.84-1.06)	.288
	Number of Exercises from Noon to 6PM	.07	1.14 (.99-1.31)	.064	.07	.96 (.84-1.09)	.291
**Interaction with Usage Days**							
	Number of Physical Health Exercises&Usage Days	.02	1.08 (1.04-1.13)	.000	.01	.99 (.98-1.01)	.571
	Number of Mental Health Exercises&Usage Days	.02	1.02 (.99-1.06)	.173	.01	.99 (.98-1.01)	.709
	Number of Nutrition Exercises&Usage Days	.01	1.01 (.99-1.04)	.256	.01	.99 (.98-1.01)	.567
	Number of Exercises from Midnight to 6AM&Usage Days	.05	1.07 (.97-1.18)	.161	.01	1.01 (.98-1.03)	.733
	Number of Exercises from 6AM to Noon&Usage Days	.01	.98 (.96-1.01)	.245	.01	1.00 (.99-1.02)	.526
	Number of Exercises from Noon to 6PM&Usage Days	.01	.97 (.95-1.00)	.043	.01	1.01 (.98-1.03)	.621
